# A new transform for the analysis of complex fractionated atrial electrograms

**DOI:** 10.1186/1475-925X-10-35

**Published:** 2011-05-12

**Authors:** Edward J Ciaccio, Angelo B Biviano, William Whang, James Coromilas, Hasan Garan

**Affiliations:** 1 Department of Medicine, Division of Cardiology, Columbia University, USA; 2 Department of Medicine, University of Medicine and Dentistry of New Jersey, USA

**Keywords:** decomposition, ensemble average, Fourier transform, reconstruction, spectral analysis

## Abstract

**Background:**

Representation of independent biophysical sources using Fourier analysis can be inefficient because the basis is sinusoidal and general. When complex fractionated atrial electrograms (CFAE) are acquired during atrial fibrillation (AF), the electrogram morphology depends on the mix of distinct nonsinusoidal generators. Identification of these generators using efficient methods of representation and comparison would be useful for targeting catheter ablation sites to prevent arrhythmia reinduction.

**Method:**

A data-driven basis and transform is described which utilizes the ensemble average of signal segments to identify and distinguish CFAE morphologic components and frequencies. Calculation of the dominant frequency (DF) of actual CFAE, and identification of simulated independent generator frequencies and morphologies embedded in CFAE, is done using a total of 216 recordings from 10 paroxysmal and 10 persistent AF patients. The transform is tested versus Fourier analysis to detect spectral components in the presence of phase noise and interference. Correspondence is shown between ensemble basis vectors of highest power and corresponding synthetic drivers embedded in CFAE.

**Results:**

The ensemble basis is orthogonal, and efficient for representation of CFAE components as compared with Fourier analysis (p ≤ 0.002). When three synthetic drivers with additive phase noise and interference were decomposed, the top three peaks in the ensemble power spectrum corresponded to the driver frequencies more closely as compared with top Fourier power spectrum peaks (p ≤ 0.005). The synthesized drivers with phase noise and interference were extractable from their corresponding ensemble basis with a mean error of less than 10%.

**Conclusions:**

The new transform is able to efficiently identify CFAE features using DF calculation and by discerning morphologic differences. Unlike the Fourier transform method, it does not distort CFAE signals prior to analysis, and is relatively robust to jitter in periodic events. Thus the ensemble method can provide a useful alternative for quantitative characterization of CFAE during clinical study.

## Background

Transforms that use a general basis like Fourier analysis are not efficient for representation of independent biophysical sources, or drivers, unless these happen to be generated by sinusoidal functions. In contrast, transforms that use data-driven bases can be efficacious for distinguishing uncorrelated signal components generated by independent drivers, if the morphology is reproduced in the basis. For example the Fukunaga-Koontz transform has been found useful to discern two independent sources in cardiac electrogram data by separating correlated versus uncorrelated components of the variance (second central moment) [1]. Development of a data-driven basis and transform that utilizes the ensemble average (first central moment) would be desirable to detect the actual signal morphologic components originating from distinct sources. This would be useful for example in the analysis of complex fractionated atrial electrograms (CFAE) [2] which are likely formed by multiple independent generators (focal areas of high frequency and/or reentrant circuits) [3-6]. Currently, CFAE are often quantified using the dominant frequency (DF), defined as the largest spectral component within the physiologic range of electrical activation rate (~2-10Hz) [7]. The DF is typically calculated by bandpass filtering the CFAE, rectification, and low pass filtering of the result, followed by Fourier power spectral analysis [8,9]. However, the filtering process distorts important signal components and the method is not robust to phase noise [10-13]. Moreover, signal morphologic components arising from each generator are not readily apparent in the sinusoidal basis. Development of an improved estimate of independent generator frequency and of morphologic characteristics would potentially be useful to target abnormal atrial tissue for catheter ablation [14], particularly for persistent AF cases [15,16].

In this study we describe a new transform which does not distort analyzed signals and is robust to phase noise, for calculation of the DF and identification of independent generator frequency and morphology in CFAE. In previous analyses of CFAE, the DF has been calculated by ensemble averaging [17,18], and this prior work was used as a foundation for development of the transform. In the current study, the transform equations are first derived. Then the transform is tested versus Fourier analysis to measure the DF of CFAE, and to determine the robustness of each method of DF measurement when random noise is added to the signal. Additionally, the frequencies of simulated drivers embedded in CFAE in the presence of phase noise and interference are detected with each method. Correspondence is shown between basis vectors of highest power derived from the new transform, versus actual CFAE morphology and synthesized drivers. Finally, DF measurement error is compared when the short-time Fourier transform and the short-time ensemble averaging transform are used to improve spectral time resolution.

## Methods

### A. Transform Equations

The autocorrelation coefficient r_φ _at lag φ is given by the inner product of two mean-zero signal vectors:(1)

where x_0 _and x_φ _are of length N and given by:(2a)(2b)

and the vectors can be normalized *a priori *by scaling to unity variance. Suppose that lag φ represents a segment of x_0 _that is w sample points long. Eq. 1 can then be rewritten as:(3)

where s_w _are segments of signal x_0 _having length w:(4a)(4b)

and the number of signal segments:(5)

Based on these equations, the autocorrelation function for all w can be described as a graph of the mean autocorrelation between successive signal segment pairs s_wi_, s_wi+1 _as given by Eq. 3, versus segment length w. The segment length can be converted to a frequency:(6)

which reduces to 1/w when the sample rate is 1 kHz and the time units are milliseconds. The peak in the autocorrelation function over a frequency range f_1 _to f_2 _(i.e., 1/w_1 _to 1/w_2_) that is physiologic for electrical activation rate has been used to estimate the DF in atrial electrograms [19-21].

A more robust alternative for adapting the autocorrelation function to spectral analysis has been developed using ensemble averaging [17,18]. The ensemble average vector e_w _is obtained by averaging the n successive mean zero segments of signal x, each segment being of length w:(7a)(7b)

where **I**_w _are w × w identity submatrices used to form the signal segments that are extracted from x and summed. Thus:(8)

where s_wi _is as given in Eq. 4a. The power in the ensemble average is described by:(9a)(9b)(9c)

where Eq. 9b and 9c are formed by substituting Eq.'s 7 and 8 into Eq. 9a, and i and j are segment numbers from 1 to n. Eq. 9c is similar to Eq. 3, except that instead of computing the autocorrelation between successive signal segment pairs s_wi_, s_wi+1 _only (lag w), it is computed between all signal segments s_wi_, s_wj_. Therefore P_w _is equivalent to computing the mean autocorrelation coefficient from n points in the autocorrelation function separated by lag w, i.e., to averaging the autocorrelation coefficients at lags w, 2w, 3w, ... nw. However, to generate P_w _in this way rather than by using Eq. 9c would require a sequence length 2N to convolve the signal with itself along its entire length, halving the time resolution and doubling the sequence length needed for analysis.

To generate the ensemble average power spectrum, the root mean square (RMS) power has been used [17,18]:(10)

which has units of millivolts. The power spectrum can be displayed by plotting √n·P_wRMS _versus frequency f as computed from Eq. 6. The √n term levels the spectral baseline, which would otherwise decrease by 1/√n, the amount of noise falloff per number of summations n used for ensemble averaging. From Eq.'s 5, 9c, and 10, the displayed RMS power can be written as:(11)

**Figure 1:**
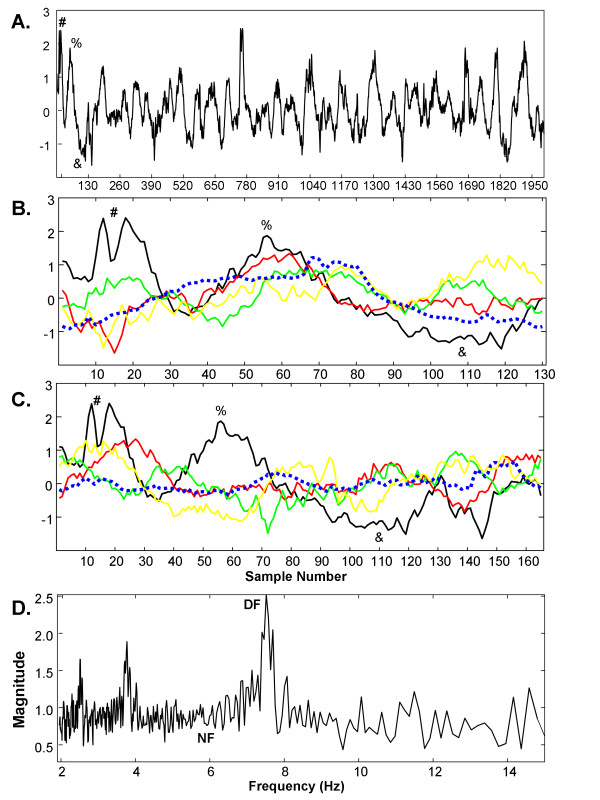
**CFAE signal and construction of its ensemble average spectrum**.

An example of ensemble average power spectrum construction is shown in Figure 1. A typical CFAE from the left inferior pulmonary vein ostia during longstanding persistent AF is shown in panel A. The summing of the first four segments of width w = 130 is shown in panel B, and they are colored black, red, green, and yellow, respectively. Some corresponding features between the CFAE trace in panel A and the first segment in panel B are noted (labeled #, %, &). The segmented traces have peaks that approximately coincide around sample numbers 55-75 (panel B). The ensemble average for all segments of width w = 130 is shown as a dashed blue trace (panel B). It has similarities to segments 1-4 shown, and to other of the CFAE segments having width w = 130 (from Eq. 5, int(8192/130) = 63 segments in total). For perspective, the x-axis scale is marked at intervals of 130 in panel A, with each scale mark representing the start of a new segment number. In panel C, segments with width w = 165 sample points are shown for comparison (fiduciary markers also labeled #, %, &). The peaks are not well aligned, and the ensemble average, again shown as a dashed blue line, is of much lower amplitude than in panel B. Thus segments with width w = 165 are not well correlated.

The ensemble average calculation was repeated for all segments w in the frequency range of interest, as given by Eq. 6, with a sampling rate of 977Hz. The RMS power in the ensemble average was then plotted using Eq. 11 and is shown in Figure 1D. The DF occurs at 7.52 Hz, corresponding to w = 130 sample points (panel B). In contrast, the location marked NF (noise floor) coincides with frequency f = 5.92 Hz, i.e., w = 165 sample points (panel C). The ensemble average spectrum thus displays correlated components as spectral peaks with higher power. These spectra also have more detail at the lower end of the range due to the w = 1/f relationship (Eq. 6), and DF subharmonics are pronounced.

The relation between the ensemble average power spectrum and the Fourier power spectrum can be described as follows. Based upon the Wiener-Khinchin theorem, the Fourier transform of the autocorrelation function of a signal is the power spectrum of that signal:(12a)(12b)(12c)

where S is the power spectral density, dφ is the phase lag w, i is the segment number, and substitution using Eq.'s 1 and 3 were utilized to form Eq.'s 12b and 12c. The Fourier power spectral density calculation decomposes the autocorrelation function into its native sinusoids. Therefore, in contrast to autocorrelation spectral analysis (Eq. 3), both ensemble and Fourier spectral analyses account for periodicity at all autocorrelation lags - ensemble by averaging (Eq. 9) and Fourier by fitting sinusoids (Eq. 12c).

The ensemble average of segments having width w is a representation of correlated signal components at the corresponding frequency and is potentially useful for signal reconstruction. From Eq.'s 7b and 9b, an ensemble average transformation matrix can be described as:(13a)(13b)

Signal x can then be decomposed using the linear transformation:(14)

where a_w _are basis vectors, n is given in Eq. 5, and a_w _and x are N × 1 in dimension. Columnwise, each identity submatrix in Eq. 13b serves to extract and sum one segment of w sample points in x (Eq. 14), with the sum total being projected onto the canonical basis. Rowwise the identity matrices serve to repeat the ensemble average of length w over a total length N during construction of a_w_. Thus the transformation matrix of Eq. 13 acts to decompose signals into periodic ensemble averages. Using the resulting basis vectors, signal x can be projected into ensemble space:(15)

where the middle and RHS in Eq. 15 are obtained by substitution and rearrangement using Eq.'s 9 and 13-14. Eq. 15 states that if each signal segment of length w is correlated with the ensemble average at w (LHS), the resulting correlation coefficient equals the ensemble average power (RHS).

In the case when N ≠ n·w above, the transformation matrix **T**_w _(Eq. 13b) must be padded by N-(n·w) rows and columns, by adding 0's as elements at the matrix's right edge, and adding clipped identity matrices as elements at the bottom edge so that the overall dimension is N × N. **T**_w _is singular for all w, since two or more rows and two or more columns are identical, i.e. it has no inverse. Thus it is not possible to transform any particular basis vector a_w _back to x, as is intuitively obvious - an ensemble average cannot be transformed back into its original signal. Suppose now that multiple transformation equations i = 1, γ are summed:(16a)(16b)

This can be rewritten:(17a)(17b)

where v is the estimate of x and  is the total transform matrix. Any two basis vectors a_i _and a_j_, i ≠ j, used for construction of v, will be orthogonal since they are formed from vectors in **T**_i _versus **T**_j _that are orthogonal, except when i/j is reducible to a small integer ratio. An example of a total transform matrix constructed from **T**_i _and **T**_j_, with dimension N = 6, is:(18)

The magnitudes are greatest along the main diagonal and equal Σ 1/n_i_, where n is given by Eq. 5. This matrix is also not invertible (Matlab ver. 7.7, R2008b, The MathWorks, Natick MA). In general, as with the individual transform matrices, the total transform matrix will not be invertible.

Consider how  acts to transform signal x. Let a subset γ of highest basis vectors, when ranked in descending order of power, be summed using  (Eq. 17). In this case  transfers the most correlated periodic components of the signal to form estimate v. The relative amplitude relationships of these components, each extracted by a different **T**_i _embedded in , are maintained by scale factor 1/n_i _during transformation (Eq. 16). However, when the components are independent (i.e., no harmonic relationships), their combination causes the 'noise' power in v to increase by √γ. To maintain the same power for best match with x, the estimate can either be scaled by 1/√γ, or alternatively v and x can be scaled to the same power. Any unique signal structure that is not periodic is also transformed by , but it is via the main diagonal, not by the off-diagonal elements which sum and reinforce correlated content. As γ is increased, the magnitude of the main diagonal elements increases so that  acts in part as an N × N identity matrix **I**_N _to directly transfer the unique uncorrelated detail during formation of v. So long as a_i _and a_j _are approximately orthogonal, the unique detail as well as correlated components maintain their correct amplitude relationships in v, since they are added in tandem and scaled by 1/n_i_.

### B. Clinical data

Atrial electrograms were recorded in a series of 20 patients, 10 with paroxysmal and 10 with longstanding persistent atrial fibrillation, referred to the Columbia University Medical Center cardiac electrophysiology (EP) laboratory for catheter ablation. Two bipolar recordings, each greater than 16 seconds in duration, were obtained from six anatomical regions: the ostia of the left superior and inferior pulmonary veins (LSPV, LIPV), the ostia of the right superior and inferior pulmonary veins (RSPV, RIPV), and the anterior and posterior left atrial free wall (ANT, POS). The recordings were obtained from these regions via the distal bipolar catheter ablation electrode during sustained AF prior to any ablation. Using standard settings, all signals were filtered in hardware at acquisition to remove baseline drift and high frequency noise (digitization rate = 977Hz; first order filter passband: 30-500 Hz). In each patient, a CFAE sequence 8192 sample points long (~8.4 seconds) as determined visually by two cardiac electrophysiologists was retrospectively selected for analysis from two sites at each of the six locations. CFAE were defined as atrial electrograms with three or more deflections on both sides of the isoelectric line, or continuous electrical activity with no well-defined isoelectric line [2]. In all, 216 of 240 recordings met these criteria, as determined by the two cardiac electrophysiologists, and were used for further analysis. No ventricular component, corresponding to the QRS deflection of the electrocardiogram, was visually evident in the CFAE. In these bipolar recordings it is uncommon for QRS artifact to be evident in CFAE obtained from the pulmonary veins and free wall. The signals were stored in both raw form, and following normalization to mean zero and unity variance.

### C. Tests of Fourier versus ensemble methods

The following six tests were developed to determine the effectiveness of the new transform versus Fourier analysis for representation of frequency and morphologic components of CFAE. The Fourier DF method is optimized when CFAE recordings are bipolar and approximately 8s in length [11,22,23]. We therefore used these parameters for most measurements in our study. The 8s sequences were readily available from retrospective data. During electroanatomic mapping, recordings with relatively short sequence length are commonly acquired from each site to minimize the procedure time.

#### C.1. Orthogonality of the ensemble basis

The inner product of normalized ensemble basis vectors was computed as:(19)

for all pairs i, j from w = 500 to w = 20 (f = 2-50Hz) for one paroxysmal and one persistent CFAE signal. The dp's were graphed for i versus j. The ensemble averaging basis was considered to be orthogonal if dp = 1.0, i = j, and dp ≈ 0, i ≠ j, except for small integer (i.e., harmonic) relationships in i/j. For comparison, dp was also calculated with the Fourier basis using the same paroxysmal CFAE signal.

#### C.2. Spectral analysis of synthetic drivers with phase noise and interference

**Figure 2:**
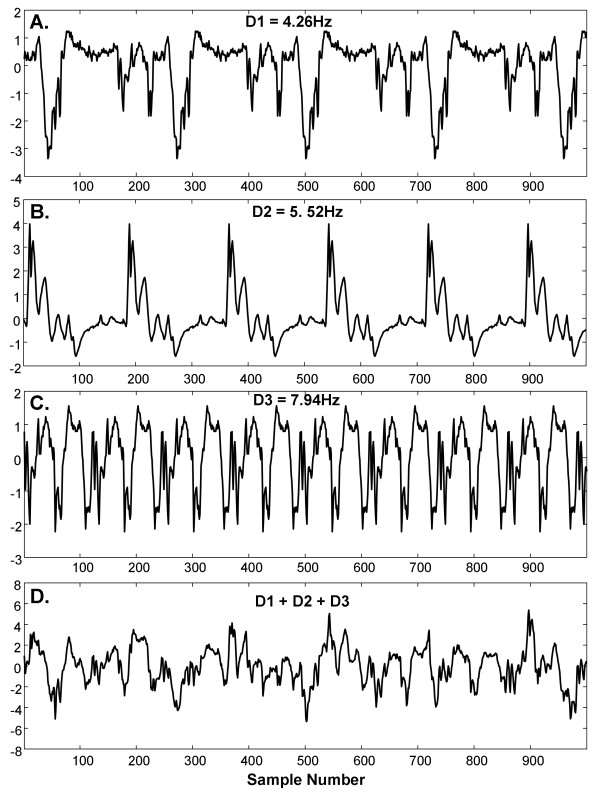
**Synthetic drivers D1-D3 and the combination D1 + D2 + D3 used for spectral analysis and reconstruction**.

**Figure 3:**
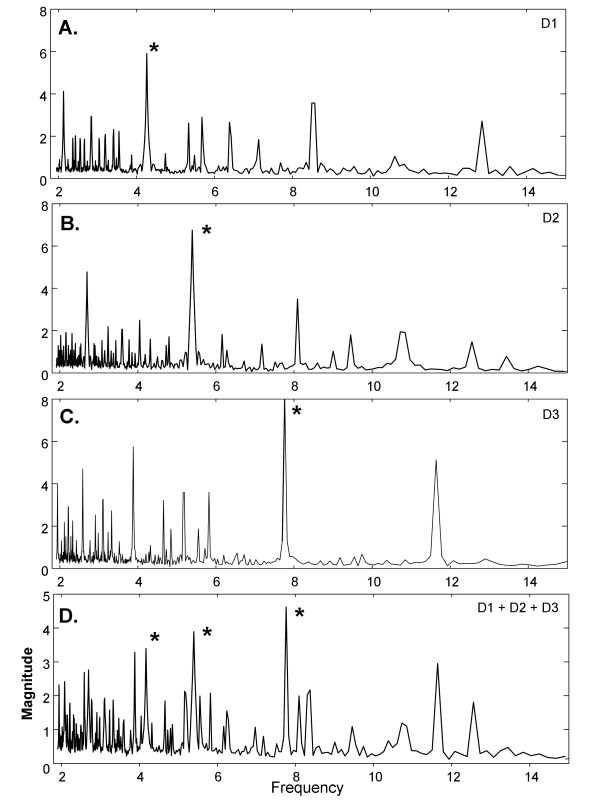
**Ensemble average spectra for the corresponding synthetic signals of Figure 2**.

Three simulated independent drivers with unrelated fundamental periodic components (DFs), were constructed from distinct CFAE deflections extracted from a single recording in one paroxysmal AF patient. The sequence lengths were 229, 177, and 123 sample points to simulate independent drivers D1, D2, and D3. Setting the sampling rate to that used for CFAE acquisition, the DFs of D1, D2, and D3 were 4.26Hz, 5.52Hz, and 7.94Hz, respectively, which is within the typical range observed in actual CFAE [2,4,7]. The synthetic drivers were normalized to mean zero and repeated to 8192 sample points. As shown in Figure 2, D1 consists primarily of downward deflections, D2 primarily of upward deflections, and D3 is biphasic. Their combination is shown in Figure 2D. The ensemble average spectra for these simulated drivers and for their sum is shown in corresponding panels of Figure 3, with DFs marked by asterisks. The harmonics of each simulated generator do not overlap.

Phase noise was created by randomly and independently shifting the timing of each driver pulse (each 229, 177, or 123 sample point interval) using a mean-zero random number generator with standard deviation of ± 16 ms. Interference was added by summing the combined synthetic signal D1+D2+D3 with one of the 216 scaled CFAE signals (i.e., the CFAE signals themselves acted as interference during measurement of synthetic driver characteristics). The following combinations of gains for the phase noise random vector (p) and interference (i) were used for assessment: (p = 1 ×,i = 1 ×), (p = 0.5 ×,i = 2 ×), (p = 0.3 ×,i = 3 ×), and (p = 0 ×,i = ± 1 ×... ± 10 ×). Fourier and ensemble power spectra were constructed in the range 2-10Hz from the resulting signals. The spectral peaks were ranked by amplitude, and the sum of ranks for peaks having frequencies of 4.26Hz, 5.52Hz, and 7.94Hz, with a tolerance of ± 0.2Hz, was tabulated. The best (minimum) sum of ranks is 6 which occurs when the driver frequencies at 4.26Hz, 5.52Hz, and 7.94Hz are ranked 1^st^, 2^nd^, and 3^rd ^in amplitude, in some combination, among all spectral peaks.

#### C.3. Identification of synthetic driver morphology

As the ensemble method but not the Fourier transform has a data-driven basis, only ensemble was used in this test. The synthetic drivers with additive phase noise and interference described in Test 2 were corrupted using two noise gain sets: p = 0.3 ×,i = 3 ×, and p = 0 ×,i = 5 ×, where the interferences consisted of the 216 CFAE signals (thus 216 comparisons for each of the two noise gain sets). The mean squared error difference between each original synthetic driver (Figure 2), and the corresponding ensemble basis vector of the corrupted signal at segment lengths of 123, 177, and 229 sample points, the periods of the drivers, when both were normalized to unity power, was tabulated in mV^2^/ms.

#### C.4. Degradation of DF in CFAE with additive random noise

This test was used to determined the efficacy of each transform to detect the DF of CFAE in the presence of random noise (no added synthetic drivers). For each of 20 selected CFAE having a prominent DF (sharp peak with its base dropping to a low noise floor), random white noise was added with a standard deviation of 0.16 mV, approximately half that of the raw CFAE signals. The absolute differences in the DF before versus after random noise addition were tabulated. This was repeated for 10 different random noise vectors. The mean and standard deviation of these values was calculated for ensemble versus Fourier spectral analysis and the significance was determined. The entire process was then repeated for random white noise with a standard deviation of 0.32 mV, approximately equal to the standard deviation of the raw CFAE signals.

#### C.5. CFAE reconstruction

The 216 CFAE recordings (no added synthetic drivers) were each decomposed and then reconstructed using 1-12 Fourier or ensemble averaging basis vectors. The mean squared error difference between each CFAE and its reconstruction from the ordered bases was determined. The reconstructions used were:(20)

where a_w1 _to a_w12 _were the top 12 basis vectors ranked in descending order of power. The average error was determined for Fourier versus ensemble reconstruction.

#### C.6. Single driver test

The CFAE signals were then altered by adding a low-power transient component at 200 sample point intervals (977 samples per second/200 samples ~5Hz). The transient itself consisted of a 42 sample point long biphasic component extracted from a CFAE acquired from the LSPV ostia during persistent AF. This transient had properties of mean = 0.13 mV, standard deviation = 0.54 mV, and peak-peak values of ~ ± 1 mV. CFAEs after addition of the low-power transient were analyzed using Fourier and ensemble spectral analysis to determine whether the component could be readily identified. Identification was defined to be presence of a distinct power spectral peak, with the base of the peak reaching the surrounding noise floor.

For tests 1-6 above, the ensemble average power spectrum was generated as described by Eq.'s 9-11 and the accompanying text. The Fortran code used for ensemble spectra calculation is provided in the Appendix and it is written to approximately halve the computation time by calculating:(21)

The Fourier power spectrum was computed using MATLAB (ver. 5.1, 1997, The MathWorks, Natick MA) by applying a Hann window to the 8192 discrete point signal. To prevent signal distortion, the traditional Fourier preprocessing method of bandpass filtering, rectification, and low pass filtering was not used (see Background). A fast Fourier transform (FFT) was then computed from the windowed signal, and the power spectrum was graphed.

#### C.7. Short-time Fourier and Ensemble Transformation

Short sequence lengths were utilized to improve time resolution of the CFAE spectral analysis. Eight successive segments of length 2048 sample points (~2s), without overlap, were extracted from each CFAE sequence. Each segment was spectrally analyzed using the Fourier and ensemble methods, and DFs were determined. The spectra were constructed as described above, except that preprocessing was included in the Fourier analysis (bandpass filtering, rectification, and low pass filtering [8,9]). The mean and standard deviation of the eight DFs were calculated from each sequence and tabulated. Suppose that measurement error in calculating DF is a mean zero random number with standard deviation α. If the actual DF is stationary, then the standard deviation σ of the measured DF will equal α. If the actual DF is nonstationary with standard deviation β, then the measured DF will have standard deviation:(22)

where the nonstationarity and measurement error are uncorrelated so that there is no divide by √n in Eq. 22. If the ensemble average and Fourier transform generate the same mean DF, then the technique with greatest standard deviation σ, regardless of whether or not the real DF is stationary, will have the largest measurement error. The means and standard deviations in 2s DF for all 216 CFAE sequences were averaged and tabulated to make this comparison.

The statistical t-test and F-test were used for detecting differences in means and variances, respectively, with significance at the p < 0.05 level (SigmaPlot ver. 9.0, Systat Software, 2004, and MedCalc ver. 9.5, MedCalc Statistical Software 2008).

## Results

This section is arranged according to the seven tests that were described in the Methods.

### A.1. Orthogonality of the ensemble basis

**Figure 4:**
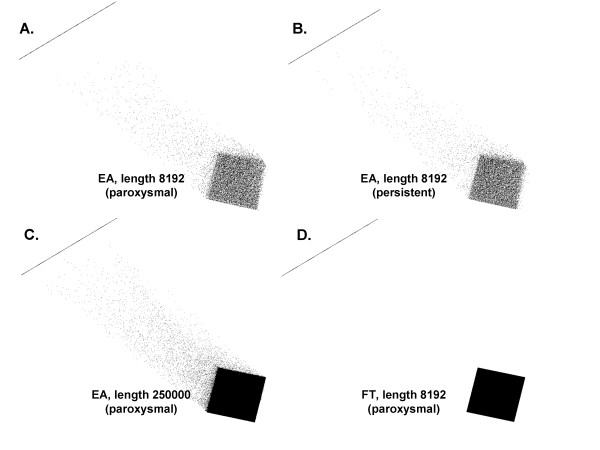
**Normalized inner product for all 481 basis vectors**. Magnitude 0 is at bottom right, magnitude 1 is at top left.

The result of the inner product measurement (Eq. 19) is shown in Figure 4, which was generated using map3d, an interactive scientific visualization tool for bioengineering data devised by the Scientific Computing and Imaging Institute, University of Utah [24]. In each panel the dp magnitude scale increases from 0 to 1 from lower right to upper left. In panels A-C the result for the ensemble method is shown, computed for all bases a_500 _- a_20 _(481 basis vectors ranging from 2Hz-50Hz). In panel A (paroxysmal AF) dp values are near zero when i ≠ j, (fuzzy square region). A line is formed at unity magnitude at upper left, corresponding to i = j (autocorrelation). Where i and j are harmonically related, the dp magnitude is intermediate (few scattered points between lower right and upper left). A similar result is obtained for the persistent AF signal (B). For all values i ≠ j including those that were harmonically related, the mean normalized inner product was 0.0075 ±.0510 for 108 paroxysmal CFAE and 0.0077 ±.0509 for 108 persistent CFAE signals (<1% of the magnitude when i = j). For N = 8192, random cancellation of uncorrelated components may have been incomplete. As a further test, the basis vectors for the paroxysmal CFAE signal were extended to N = 250,000 in length, and the resulting inner products are graphed in panel C. In this panel when i ≠ j and no harmonic relationship exits, dp = 0.0 (square region is solid rather than fuzzy i.e. there is complete cancellation of random components). Thus the ensemble basis is orthogonal except for small integer harmonic relationships. For comparison, the dp using Fourier bases (N = 8192) is shown in panel D. Since the sinusoidal basis is antisymmetric about the x-axis, the inner product is zero when i ≠ j, even for harmonic relationships.

### A.2. Spectral analysis of synthetic drivers with phase noise and interference

**Figure 5:**
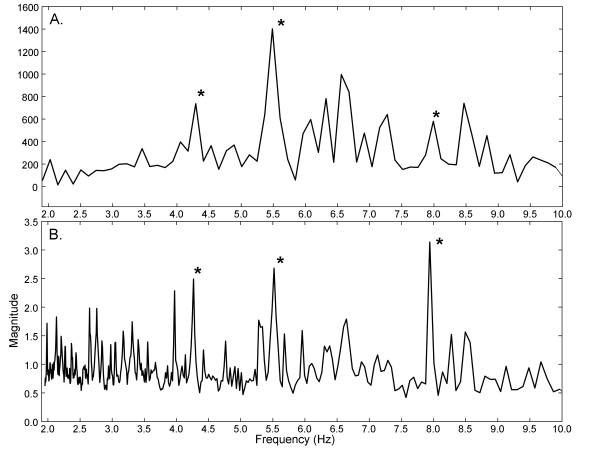
**Comparison of power spectra**. A. Fourier spectrum. B. Ensemble spectrum. Noise level: p = 0 ×, i = 5 × (i.e., no phase noise, interference added with 5 × gain).

In Figure 5A-B are shown Fourier and ensemble average spectra of the three synthetic drivers when interference is added (p = 0 ×, i = 5 ×). Most of the spectral components are caused by the drivers, with the interference contributing to the noise floor (compare Figure 5B and 3D from 2-10Hz). The location of synthetic driver peaks are noted by asterisks. Portions of the noise floor extend beyond two driver peaks in the Fourier spectrum (Figure 5A). In contrast, the driver peaks are all higher than the noise floor for the ensemble average spectrum (Figure 5B). The overall result for measurements with the various additive noise combinations and interferences is shown in Table 1. In the first and second columns are noted the phase and interference multipliers, respectively. In the third and fourth columns are noted mean ± standard deviation in the sum of ranks for D1, D2, and D3. The significance of the differences are noted in the last two columns. All of the means are significantly different, with the synthetic drivers being more highly ranked in the ensemble average spectra (total rank is closer to 6). The standard deviation in total rank, i.e. the variability in detecting the driver peaks, is larger in Fourier as compared with ensemble averaging, with a significant difference in two cases.

**Table 1:** Sum of Ranks of Three Driver Frequencies

p	i	Fourier (Hz)	Ensemble (Hz)	Significance MN	Significance SD
1 ×	1×	7.12 ± 1.41	6.71 ± 1.08	.005	NS

.5 ×	2 ×	7.03 ± 0.48	6.31 ± 0.10	<.001	<.001

.3 ×	3 ×	7.88 ± 0.30	6.73 ± 0.08	<.001	<.001

0 ×	±10 ×	10.24 ± 3.37	8.82 ± 3.08	<.001	NS

p = gain of added phase noise. i = gain of added interference.

Significance MN = significance of the difference in mean values using the unpaired t-test

Significance SD = significance of the difference in the standard deviation from the mean using the F-test.

NS = not significant

### A.3. Identification of synthetic driver morphology

**Figure 6:**
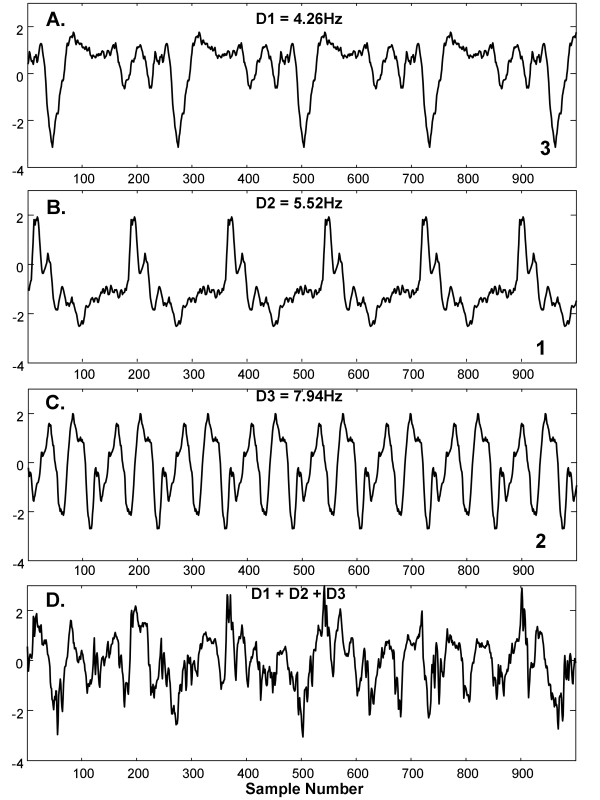
**A-C. The top three ensemble basis vectors constructed from a synthesized signal with phase noise added having 0.3 × gain, and interference having 3 × gain**. D. The sum of these basis vectors. The drivers from which the synthesized signal was constructed are shown in Figure 2.

In Figure 6 is shown an example of the top three basis vectors (panels A-C) constructed from synthetic drivers after addition of phase noise and interference (weighting p = 0.3 ×, i = 3 ×, panel D). The basis vectors in Figure 6A-C and the combination in 6D are reflective of the corresponding drivers of Figure 2. Some smoothing occurs in the fine detail due to the phase noise (jitter) that was added to the drivers. The 4.26Hz, 5.52Hz, and 7.94Hz bases were ranked the 3^rd^, 1^st^, and 2^nd ^highest peaks, respectively, in the ensemble averaging power spectrum, as is noted at bottom right in each panel A-C. For the noise set (p = 0 ×, i = 5 ×) the corresponding basis vectors estimated the Figure 2 drivers almost exactly, as there was no added jitter (not shown). For 216 tests with phase noise and interference (p = 0.3 ×, i = 3 ×) the average mean squared error was 0.091 ±.020mV^2^/ms while for additive interference only (p = 0 ×, i = 5 ×), it was 0.0049 ±.0042mV^2^/ms. These errors are <10% of the power in the normalized drivers (1.0 mV^2^/ms). Thus in the presence of jitter and/or interference, morphologic components of independent drivers in CFAE are extractable using the ensemble basis.

### A.4. Degradation of DF in CFAE with additive random noise

**Figure 7:**
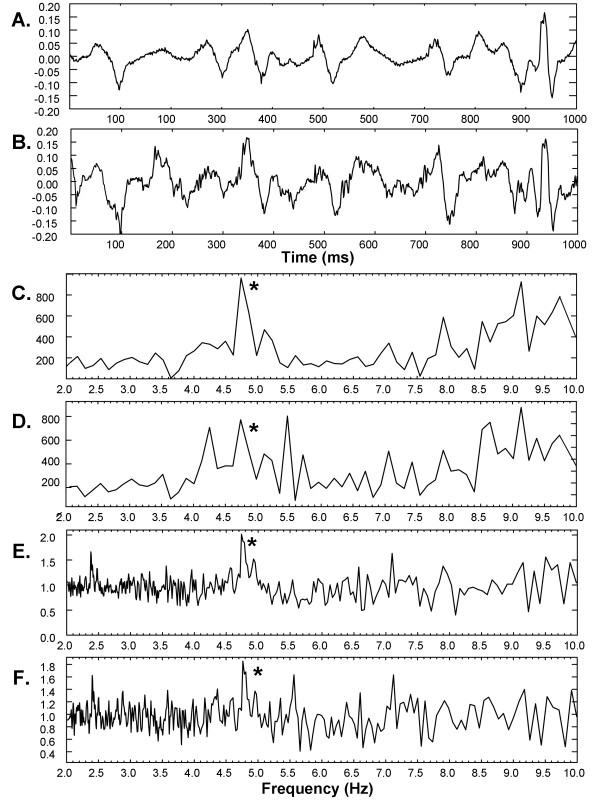
**A. CFAE from a paroxysmal AF patient - anterior left atrial free wall**. B. The CFAE with random noise added. C-D. The Fourier power spectrum for the signals of panels A-B, respectively. E-F. The ensemble power spectrum for the signals of panels A-B, respectively.

For random noise added with SD = ± 0.16 mV, the mean absolute difference in DF before versus after addition of a random noise vector was 0.35 ± 0.02Hz for Fourier spectral analysis versus 0.09 ± 0.05Hz for ensemble spectral analysis (p < 0.001). For random noise added with SD = ± 0.32 mV, the mean absolute difference in DF before versus after addition of a random noise vector was 0.68 ± 0.10Hz for Fourier spectral analysis versus 0.53 ± 0.13Hz for ensemble spectral analysis (p = 0.01). An example is shown in Figure 7 for a CFAE signal from the anterior left atrial free wall of a paroxysmal AF patient. Panels 7A-B show the CFAE prior to and after addition of random noise with SD = ± 0.16mV, while panels 7C-D and 7E-F show the corresponding Fourier and ensemble average spectra. In each spectrum the DF is noted by an asterisk. After noise addition, the DF peak is only the third highest in the Fourier spectrum (panel D) but it remains the highest peak in the ensemble average spectrum (panel F). Thus as shown by Figure 7 and Table 1, the DF peak in ensemble spectral analysis is more robust to addition of random white additive noise such as might occur in a clinical setting due to presence of motion artifact, electrical noise, and/or broken wire leads.

### A.5. CFAE reconstruction

**Figure 8:**
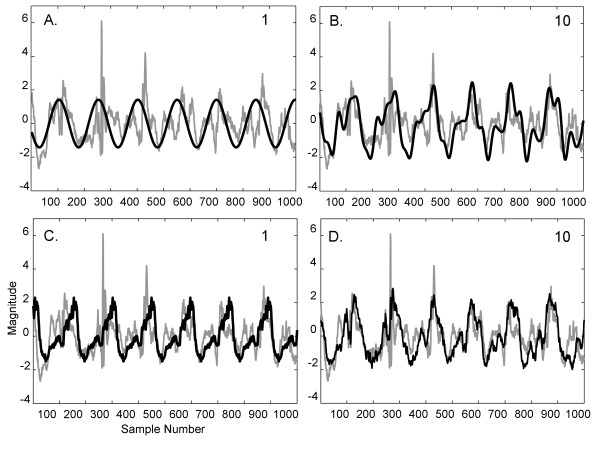
**CFAE reconstruction with 1 and with 10 ordered basis vectors is shown for: A-B**. Fourier analysis and **C-D**. ensemble analysis.

An example of the Fourier basis vectors a_w _constructed from the ensemble averages e_w _with 1^st ^and 10^th ^highest power is shown in Figure 8A and 8B from a paroxysmal CFAE signal acquired from the LIPV ostium. The corresponding ensemble averaging basis vectors for this same signal are shown in Figure 8C and 8D with scales of corresponding axes the same as in 8A-B. As the Fourier basis is general and sinusoidal, the estimates approximate the signal with relatively large error (8A and 8B). However, as the ensemble averaging basis is data-generated and constructed from the first central moment of the signal, it is more estimative of the CFAE even when only the single most important basis vector is used (Figure 8C). There is substantial overlap with the actual CFAE trace when 10 basis vectors are used for reconstruction (Figure 8D). For all 12 reconstruction vectors combined, the root mean square error averaged 1.13 ± 0.07mV for Fourier versus 0.98 ± 0.10mV for ensemble (p < 0.001). The reconstruction error was lower for ensemble averaging versus Fourier for each individual reconstruction using 1 (p = 0.002) and 2-12 bases (p ≤ 0.001).

**Figure 9:**
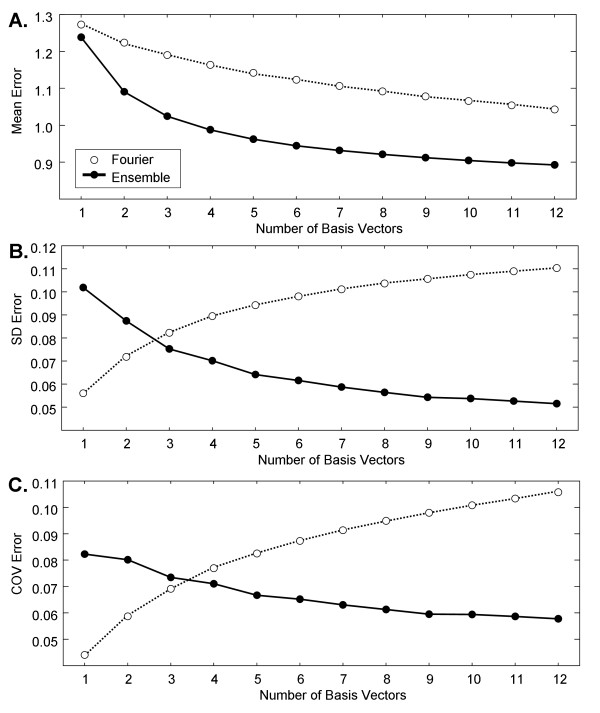
**The statistics of Fourier and ensemble average reconstruction error for real CFAE signals**.

The statistical relationships are illustrated in Figure 9. The mean error in reconstruction for ensemble averaging decreases more rapidly as compared with Fourier (Figure 9A). The standard deviation in the reconstruction error for all CFAE is shown in Figure 9B. The standard deviation falls off rapidly for ensemble averaging and increases rapidly for Fourier. At ≥3 basis vectors, the standard deviation in reconstruction error is lowest for ensemble averaging. This means that the ability of ensemble averaging to consistently reconstruct CFAEs (panel 9B) with a relatively low level of error (panel 9A) is mostly improved as compared with Fourier reconstruction. Similarly, the coefficient of variation, which is the standard deviation divided by the mean (Figure 9C), falls off for ensemble average reconstruction but it actually increases for Fourier reconstruction.

### A.6. Single driver test

**Figure 10:**
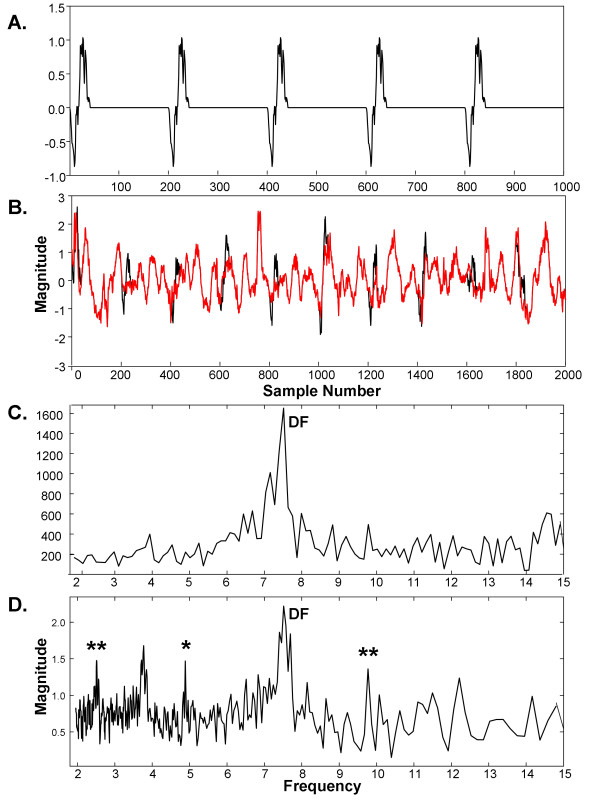
**Illustration of the effect of a transient on a CFAE signal**. A. Transient. B. CFAE from left inferior pulmonary vein of a persistent AF patient (red). The CFAE with transient added is shown in black. C. Fourier power spectrum of the signal in panel B. D. Ensemble average power spectrum of the signal in panel B.

**Figure 11:**
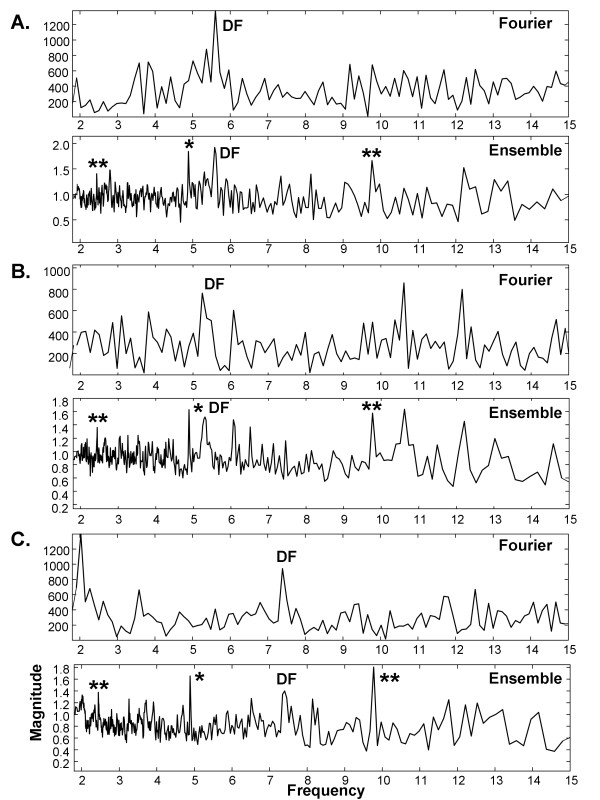
**Additional examples of Fourier versus ensemble average power spectra with the transient added to CFAE signals**. Transient peak is marked by * and harmonics by **; DF is labeled. A. paroxysmal AF, anterior left atrial free wall, B. persistent AF, left inferior pulmonary vein ostia, C. paroxysmal AF, left inferior pulmonary vein ostia.

The 5Hz transient described in the Methods is shown in Figure 10A and its addition to a CFAE is shown in Figure 10B, black trace. For comparison, the original CFAE is shown as the red trace in panel B and it is the same trace as in Figure 1A. The Fourier and ensemble average power spectra are shown in Figure 10C and 10D, respectively. Although both spectra show a DF at ~7.5Hz and a smaller peak at ~3.9Hz (which may be generated by an independent driver), only the ensemble average power spectrum indicates presence of the artificial transient at 5Hz (noted by *; with super- and subharmonics noted by **). For all CFAEs, the 5Hz transient was identified in 216/216 ensemble average spectra (100%) but was only present in 82/216 Fourier spectra (38.0%). Additional examples are provided in Figure 11. In each pair of Fourier and ensemble spectra, both have the same DF in the range 3-10Hz. However, the 5Hz transient is evident only in the ensemble averaging spectra (again noted by *; with super- and subharmonics noted by **). Thus ensemble averaging but not Fourier spectral analysis is sensitive to the presence of far-field and/or low-power drivers which affect CFAE over short intervals.

### A.7. Short-time Fourier and ensemble transformation

The averages for mean and for standard deviation in 2s DF over 216 sequences are shown in Table 2. For both Fourier and ensemble analysis, mean DF is ~5.2Hz for paroxysmal versus ~6.4Hz for persistent AF (p < 0.001, row 4). There were no significant differences between the mean DFs as measured by Fourier versus ensemble. The 2s standard deviations in DF are shown in the right-hand columns, Table 2. The difference in these values between paroxysmal and persistent AF patients were not significant (NS, row 4). The 2s standard deviations were lower for ensemble as compared with Fourier (right-hand columns). Although this difference was not statistically significant, it suggests a lower measurement error for ensemble as compared with Fourier spectral analysis when DF is computed using sliding, non-overlapping 2s windows to improve time resolution (see Eq. 22 and accompanying text).

**Table 2:** Comparison of Short-Time Transform Frequency Measurements

Type	Mean_FT	Mean_EA	SD_FT	SD_EA
Paroxysmal, DF (Hz)	5.18	5.25	0.62	0.61

Persistent, DF (Hz)	6.39	6.35	0.55	0.50

Significance	p < 0.001	p < 0.001	NS	NS

Combined	5.78	5.82	0.44	0.41

DF = dominant frequency, FT = Fourier transform, EA = ensemble average analysis, SD = standard deviation.

## Discussion

### A. Synopsis

In this study a data-driven transform was described for application to CFAE signals. The basis is constructed from the ensemble averages of signal segments and was found to be orthogonal except for small integer-multiple (i.e. harmonic) relationships. The power in each ensemble average is equivalent to the projection of the signal onto the corresponding basis (Eq. 15). The relationship of the ensemble average spectrum to the autocorrelation spectrum and to the Fourier power spectrum was described. While the autocorrelation spectrum is based on correlation at a single lag w, the ensemble and Fourier power spectra are based on correlation at multiple lags w, 2w, ..., nw. During construction of the ensemble spectrum, the autocorrelation function at lags are averaged, as compared to the Fourier power spectrum which is a sinusoidal curve-fitting of the autocorrelation function. Several tests were used to compare the efficacy of the Fourier transform, versus transformation using ensemble averaging, for representation of CFAE signal components. At several levels of additive noise and interference, the highest peaks in the ensemble spectrum corresponded to the frequencies of three synthetic drivers with greater accuracy as compared to Fourier spectral analysis (p ≤ 0.005; Table 1). Similarly, when random white noise corrupted actual CFAE signals, the ensemble spectrum was more accurate than Fourier in representation of the DF (p ≤ 0.01). The ensemble basis was found to be useful for representation of the signal morphology of three independent synthetic drivers. When only interference was added to the combined driver signal, the three top-ranked basis vectors in order of greatest power corresponded to the independent driver morphology almost exactly. When phase noise (jitter) was added, the three top-ranked basis vectors corresponded to driver morphology but with some smoothing. When a single low-power, short duration component was added, as would simulate presence of a distant driver, it was evident as a distinct peak in all 216/216 ensemble average spectra but in only 82/216 Fourier spectra. During reconstruction of actual CFAE signals, the ordered ensemble average basis from 1-12 vectors was more accurate for representation as compared with Fourier (p ≤ 0.002). Finally, DF measurement error as estimated by the standard deviation in DF was reduced in ensemble as compared with Fourier spectral analysis when short-time transformation was used to improve time resolution. Thus it was found that the new transform is efficacious for representation and measurement of morphologic and frequency components in real CFAE, and in synthetically-derived independent generators, as compared with the Fourier transform.

### B. Computational and mathematical considerations

Although ensemble average analysis is relatively robust to noise and jitter, to further reduce their affect on signal analysis, the inner product between the spectrum and a model can be used for gradual, adaptive update [25] or alternatively, finite differences can be used for adaptation [26]. When computing the DF of atrial fibrillation signals, variation by as much as 2.5Hz can occur over a time interval of a few seconds; hence tracking with time-frequency methods may be required for accurate analysis [27,28]. Since ensemble averaging is a form of autocorrelation, a minimum sequence length of two cycles of the periodic signal is needed for construction of the frequency spectrum (which would result in a very course estimation). To include low frequency activity to a lower limit of 2Hz, as was done in this study, a window of at least 1000ms (1s) should thus be used. Any such measurement could be updated by shifting the analysis window, for example by 100-150ms steps, to describe the time-frequency evolution of the signal [29]. To reduce error when short sequences are utilized for analysis, a model-based approach for update of the spectral profile can be implemented [30].

In a study by another group, the DF computed by Fourier analysis was compared with the mean, median, and mode activation rate, as obtained by electrogram marking, to determine efficacy [10]. However, as stated in that study, DF does not specifically reflect activation rate and therefore is only an approximate measure, with a level of uncertainty. For this reason, we chose to add artificial drivers at specific frequencies, as well as to analyze the degradation of actual DFs in CFAE when random noise is introduced, as tests to compare the Fourier versus ensemble averaging methods. In each of our tests of DF measurement, the highest peak in the spectral range was selected as the DF. The more accurate selection of DF in presence of noise and interference by ensemble average analysis may in part be due to increased spectral power in the fundamental frequency versus the superharmonics as compared with the standard Fourier method [18].

### C. Clinical applications and future directions

Knowledge of the mechanisms for onset and maintenance of atrial fibrillation is scant due in part to the difficulty in quantitative assessment of the CFAE signal with the standard Fourier method of DF calculation, which distorts the signal during preprocessing and suffers from phase noise degradation of the estimate [31]. By devising a data-driven frequency transform, independent synthetic drivers were successfully extracted and characterized using both frequency and morphologic measurements in our study. It is anticipated that independent drivers can similarly be discerned in real clinical data. For example, when a simulated mother rotor located in a uniformly conducting medium drives AF, around which is a boundary leading to nonuniformly conducting regions, then the rotor's frequency components will still be prominent in nonuniform region spectra, although the noise floor increases [32]. At such conduction boundaries, jitter and additive random noise caused by wavelet formation likely act to increase the noise floor. Based upon the results of this study, ensemble spectral analysis is more robust to jitter and random noise in CFAE as compared with Fourier, and thus is probably more likely to identify distant drivers even in nonuniformly conducting regions where secondary wavelets form. Such uniform/nonuniform boundaries can be quite common in arrhythmogenic atrial tissue, and include interfaces between electrically remodeled versus nonremodeled tissue, and between areas with fibrosis versus no fibrosis.

The transform can be further developed for clinical use by activation mapping of the substrate during clinical electrophysiologic study, identification during AF of independent focal or reentrant sources in the maps, and determining the correspondence of these to the most important ensemble average basis vectors and frequency components. It is expected that ablation lesions at these sources will prevent AF [5,6], although this must be verified. Simulations have suggested that sinusoidal electric fields may be important for excitation of cardiac tissue [33]. If such sinusoidal generators exist in nature, they will be efficiently represented by the Fourier transform, which is based upon sinusoidal components, but also by the ensemble averaging basis, from which any such components are also readily reconstructed.

This study was limited to retrospective analysis of sequentially acquired CFAE, from which the ensemble averaging method was compared with the Fourier transform. Future clinical research is planned to project the AF signals into ensemble averaging space using Eq. 15, for solution of two- and multiple-class problems. A plot of x^T ^· a_w _versus w is the ensemble power spectrum. One way to express differences between CFAE would be based upon the Euclidean distance in ensemble average space:(23)

where P and P' denote power spectra of two different CFAE. Suppose for example that many CFAE recordings are obtained simultaneously from the left atrium. The ensemble spectrum of each can be compared with its nearest-neighbors by averaging the Euclidean distance between nearest-neighbor spectra. If this is repeated for nearest-neighbors throughout the mapping region, then areas with smallest Euclidean distance would suggest presence of a nearby driver or substrate homogeneity, while areas with large spectral difference would suggest either locally heterogeneous properties of electrical conduction, or a boundary area where multiple independent drivers compete. Another method of classification would be to sum all CFAE in a neighborhood region, compute the ensemble average basis, project each CFAE onto the ensemble average space resulting from this basis, and cluster and classify according to the position of each point in the space.

Our study was limited to the measurement of retrospectively-obtained CFAE. According to our results, under certain conditions, ensemble averaging is more robust to phase noise and interference as compared with Fourier analysis. However, to show that the new method is efficacious for clinical electrophysiologic study, CFAEs of AF patients should be classified prospectively into ablative and non-ablative groups, followed by catheter ablation at the areas designated for ablation. The efficacy would then be determined by any reduction in procedural time, and in the need for follow-up study, as compared with not using the technique.

The importance of the ensemble averaging method will also depend upon other practical applications. In recent work, DF maps at early onset of ventricular tachyarrhythmia in postinfarction canine border zone were constructed with ensemble averaging [29]. Using DF gradients, it was possible to predict the arrhythmia type that would be manifested (monomorphic ventricular tachycardia, polymorphic ventricular tachycardia, or ventricular fibrillation). This technique could be useful for arrhythmia detection from an ICD at a time sufficiently early in onset so that low voltage shock would prevent ventricular fibrillation [29], the subject of future study. In another recent study, videocapsule endoscopy images were assessed with ensemble averaging for estimation of the dominant period over many image frames [34]. This dominant period was found to be correlated to small bowel motility, which can be altered in celiac disease and in patients with other diseases causing small intestinal lesions. Furthermore, ensemble average spectra have been used to show differences at the pulmonary vein ostia and the left atrial free wall in paroxysmal as compared with longstanding persistent AF patients [35]. Using ensemble spectra, the study showed that there is greater spatial uniformity of left atrial DF in longstanding persistent as compared with paroxysmal AF patients. The DF also tended to approach a common upper bound value of ~6.0 - 6.5Hz in persistent AF patients [35]. Thus the new transform may have wider application for prospective clinical data analysis.

### D. Limitations

The frequency decompositions used in this study are strictly applicable only to stationary signals. Thus caution must be exercised in interpreting the spectra as being representative of the entire measured interval. When nonstationarity is present, other transforms such as Wavelet decomposition can provide additional information. For some comparative tests, three synthetic drivers were constructed by extracting sequences from one CFAE. In another test, a short low-power transient was synthetically added to CFAE to simulate a far-field driver. To confirm the utility of the new transform, tests with synthesized data should be repeated using real AF drivers as determined by activation mapping (noncontact or sequential contact mapping). Confirmation of the reduction in error in the decomposition and reconstruction of CFAE signals by ensemble averaging as compared with other transformations, and in its robustness to noise, may require proving optimality, the subject of future research. The frequency resolution of the ensemble average spectrum can be made homogeneous by using appropriate fractional values of w and interpolating between sample points of the signal, although for simplicity it was not done in this study.

## Conclusions

We have described a new transform that can be used for evaluation of CFAE signals. The ensemble average of signal segments was utilized to construct a data-driven basis, and it was shown to have significant advantages over Fourier analysis for correct prediction of the DF of independent synthetic drivers in the presence of phase noise and interference, identification of the distinctive morphologic components associated with each driver, as well as for representation of CFAE signals in general. The transform therefore may have application to prospectively target atrial fibrillation drivers during clinical catheter ablation to prevent arrhythmia recurrence, as well as for improved understanding of the mechanisms by which paroxysmal and persistent AF are initiated and maintained.

## Competing interests

The authors declare that they have no competing interests.

## Authors' contributions

EJC developed the mathematical transform and conducted the data analysis. ABB, WW, JC, and HG made helpful suggestions for comparison of the new transform with Fourier analysis, provided the clinical data, and determined which recordings were CFAE. All authors have read and approved the final manuscript.

## Appendix

The following tested Fortran code is useful to compute ensemble average power spectra from multiple CFAEs. The code runs in ~1 second on a PC-type laptop computer and can be implemented in real time.

   parameter (n0 = 50, n1 = 500, n2 = 8192, n3 = 216, rate =.977)

   real en(n1, n1), f(n1), inp(n2, n3), s(n1, n3)

   do 1   i = 1, n3

      do 2   j = n1/2+1, n1

         en(j, 1:j) = 0.

         do 2   k = 1, n2/j

            en(j, 1:j) = en(j, 1:j) + inp((k-1)*j+1:(k-1)*j+j, i)

2   continue

      do 3   j = n1/2, n0, -1

         en(j, 1:j) = en(2*j, 1:j) + en(2*j, j+1:2*j)

3   continue

      do 1   j = n0, n1

         s(j, i) = sqrt(sum(en(j, 1:j)**2)/n2); if(i.eq.1) f(j) = rate/j

1   continue

en = ensemble vector, rate = digital sampling rate, s = spectral magnitude, f = frequency

inp = input matrix consisting of CFAEs normalized to mean zero and standard deviation = 1.

n0, n1 = range of segment widths = (50, 500). Frequency f = 977/50 - 977/500 = 19.54Hz - 1.95Hz.

n2 = number of sample points in each CFAE = 8192.

n3 = number of CFAE from which to calculate spectra = 216.

Loop 1 (inner) computes the RMS power spectrum s(j, i) based upon Eq. 11, with frequencies given by f.

Loop 2 zeros the ensemble matrix and computes ensemble averages from (n1)/2 + 1 to n1.

Loop 3 computes ensemble averages from w = n0 to (n1)/2 by averaging the two half segments of the ensemble average with width 2w (see Eq. 21).
